# Evidence of Tree Species’ Range Shifts in a Complex Landscape

**DOI:** 10.1371/journal.pone.0118069

**Published:** 2015-01-29

**Authors:** Vicente J. Monleon, Heather E. Lintz

**Affiliations:** 1 Pacific Northwest Research Station, United States Forest Service, Corvallis, Oregon, United States of America; 2 Oregon Climate Change Research Institute, College of Earth, Ocean, and Atmospheric Science, Oregon State University, Corvallis, Oregon, United States of America; INRA - University of Bordeaux, FRANCE

## Abstract

Climate change is expected to change the distribution of species. For long-lived, sessile species such as trees, tracking the warming climate depends on seedling colonization of newly favorable areas. We compare the distribution of seedlings and mature trees for all but the rarest tree species in California, Oregon and Washington, United States of America, a large, environmentally diverse region. Across 46 species, the mean annual temperature of the range of seedlings was 0.120°C colder than that of the range of trees (95% confidence interval from 0.096 to 0.144°C). The extremes of the seedling distributions also shifted towards colder temperature than those of mature trees, but the change was less pronounced. Although the mean elevation and mean latitude of the range of seedlings was higher than and north of those of the range of mature trees, elevational and latitudinal shifts run in opposite directions for the majority of the species, reflecting the lack of a direct biological relationship between species’ distributions and those variables. The broad scale, environmental diversity and variety of disturbance regimes and land uses of the study area, the large number and exhaustive sampling of tree species, and the direct causal relationship between the temperature response and a warming climate, provide strong evidence to attribute the observed shifts to climate change.

## Introduction

Climate change is predicted to cause systematic changes in the geographic distribution of species. A recent meta-analysis of 23 studies estimating shifts in latitude and 31 estimating shifts in elevation reported an overall migration rate of 16.9 km poleward and 11.0 m upward per decade [1]. While evidence supports that such changes are occurring, the estimation of the magnitude of change and the attribution of cause are challenging tasks, with the strength of evidence increasing as the geographic area, number of species, and length of time examined increases [2, 3]. Attribution can also be complicated because most studies examine shifts in either latitude or elevation, but those variables are surrogates for other environmental drivers, primarily temperature, and neither variable has much biological meaning per se [2, 4]. Further, because both latitude and elevation affect temperature, estimating the effect of each variable separately, without accounting for the other, may result in spurious relationships. Many studies, particularly those examining elevation shifts, are conducted in a single location [5, 6], effectively controlling for the effect of latitude on elevation. However, reducing the geographic extent to a single site also reduces the generality of the results and complicates attribution [3]. Observed changes could be caused by local effects that covary with elevation, such as disturbance, local drought or changes in land use, instead of by changes in temperature [7, 8]. For large-scale studies [9, 10], where both latitude and elevation vary, confounding between those variables can be very significant and mask effects of climate warming. For example, lack of northward change in the southern boundary of the range of many species in Europe can be explained because they reach their southern limit in mountain ranges. Therefore, species may have responded to climate warming by migrating upwards within the mountains, rather than northwards [11]. A species can track increasing temperatures by shifting its range upwards, polewards, or a combination of both. The relative magnitude, or even direction, of those changes may depend on a number of factors, including the species’ ecological traits and the geographic distribution of suitable habitat in the region.

Estimating the magnitude of range shift is also complicated because most studies rely on haphazardly or purposively selected samples, often comparing contemporary data with historical datasets, rather than on a probability sample from a well-specified population. Inference from those samples requires strong assumptions about the representativeness of the sample or the behavior of the underlying population. Estimators may reflect the probability of unit selection into the sample instead of estimating the population parameters of interest [12]. The likelihood of spurious relationships would increase if the sampling intensity is correlated with a confounding variable, as would be the case, for example, if contemporary and historic samples are compared, but they differ in their latitude or elevation distribution. Thus, reported downhill shifts of plant species in California [10] have been questioned, because the contemporary sample was much north of the historic sample [13–15]. Many studies select samples to minimize direct anthropogenic impacts that could mask the climate change effects, for example selecting high elevation sites [16], protected or inaccessible areas [6], or discarding plots with signs of disturbance [9]. However, restricting the sample also limits the scope of inference of the study. Migration rates estimated from those samples may not reflect the actual species’ response across its range, where other effects such as habitat change or fragmentation may limit their ability to track climate warming [11, 17, 18].

**Fig 1:**
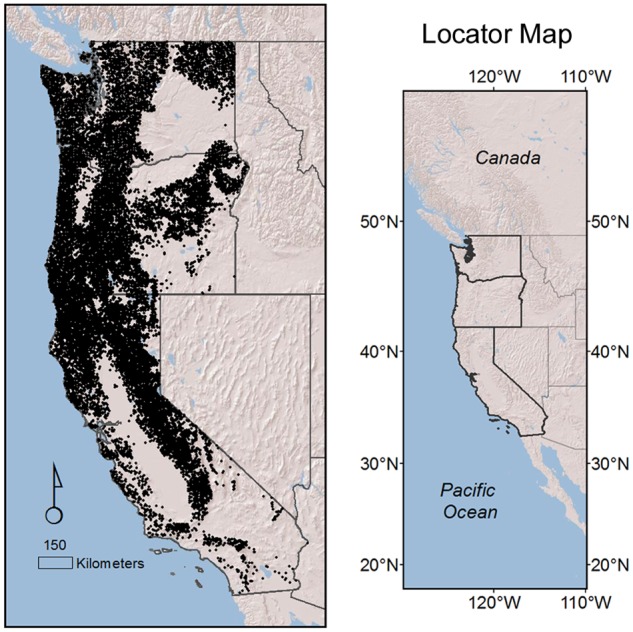
Study area with the location of the 14,105 forested plots. The total area is 823,000 km^2^, 42% forested.

In this study, we estimate changes in the distribution of all but the rarest tree species across all forestlands of the U.S. Pacific coast states, a large, environmentally diverse and floristically rich region (Fig. 1). We base our estimates on a spatially balanced, probability sample of the entire population. This sampling design ensures that there is no sampling selection bias and allows for model free, approximately unbiased estimation of change in the mean and quantiles of the species’ distributions [19]. The sampling design allows the joint examination of shifts in elevation and latitude, thus assessing the potential effects of confounding between those two variables.

Repeated measurements of the same plots at different point in time were not available. However, range shifts ultimately depend on changes in the rates of recruitment and mortality in different regions of the species’ range. Thus, as a surrogate for range shift over time, we compare the current distribution of seedlings to that of mature trees. A warmer climate would drive seedling establishment towards colder areas relative to areas occupied by mature trees, which reflect past recruitment. The environmental requirements of seedling and mature trees may be somewhat different [20], but this approach has been used to study vegetation dynamics and succession (e.g., [21]) and the response of trees species to climate warming [22–26]. The presence of seedlings is certainly a necessary condition for the future presence of mature trees and, therefore, is at least an indicator of potential change. Further, for long lived, sessile species such as trees, mature individuals may persist in the landscape even when recruitment is limited or non-existent, delaying the manifestation of range shifts [27] and weakening the relationship between current species distribution and climate [26, 28]. We are, however, implicitly assuming that the distribution of mature trees has remained relatively stable in the recent past, an assumption that may not hold for a species that has suffered episodes of widespread mortality [29].

The specific objectives of this study are to estimate the difference in mean latitude, elevation and annual temperature of the range of seedlings and mature trees across all forestlands of California, Oregon and Washington, U.S.A.

## Materials and Methods

### Study Area

The study includes all forestlands in California, Oregon and Washington, USA, an environmentally diverse and floristically rich region. Latitude ranges from 32° 32’ to 49° N, spanning 1,850 km. Elevation ranges from 86 m below to 4,421 m above sea level. Total land area is 823,091 km^2^, of which an estimated 345,060 km^2^ is forestland [30]. Forest land is broadly defined as an area greater than 4,050 m^2^ with at least 10 percent potential stocking with tree species, and excluding urban and agricultural land uses [31, 32]. Trees are plant species able to reach a height of 5 m at maturity. The study area includes 19 ecoregions [33] and many distinct forest types, including warm and cold deserts, semi-arid woodlands, montane and high elevation forests, and temperate rain forests. Approximately 13% of the forest land is in reserved areas, with the remaining evenly divided between public and private ownership [30]. Management regimens vary widely, from wilderness areas to intensively managed, short-rotation plantations.

### Sampling and Measurement Design

The study is based on datasets from the US National Forest Inventory. The field data was collected by the US Forest Service Forest Inventory and Analysis Program (FIA) and did not involve any field data collection by any of the authors. The databases are available at www.fs.fed.us/pnw/rma/fia-topics/inventory-data/. However, the Food Security Act of 1985 protects the confidentiality of the plot location to ensure the privacy of the landowners and protect the integrity of the sample. Users needing the exact plot coordinates may contact the FIA program directly at www.fia.fs.fed.us/tools-data/customer-service/. Obtaining the exact plot coordinates requires approval from the FIA program and a Confidentiality Agreement.

The sampling design consisted of a spatially balanced, probability sample of one ground plot every 24 km^2^ in California and Oregon (measured between 2001 and 2010) and every 26.6 km^2^ in Washington (measured between 2002 and 2010). The total sample size was 33,674 plots, of which 14,105 were forested and 1,684 could not be measured, either because the landowner denied access, or because the plot was unsafe to reach or occupy. Plots were a cluster of 4 points within a 1 ha circle. If a plot contained forest land, it was installed and measured. At each of the 4 points, trees with stem diameter greater than or equal to 12.7 cm were tallied in a 7.32 m radius circular subplot (total area 672.5 m^2^). Trees with stem diameter greater than or equal to 2.54 cm, but less than 12.7 cm, were tallied in a 2.07 m radius circular subplot (total area 54 m^2^). Seedlings, defined as trees with stem diameter less than 2.54 cm and length greater than or equal to 15.2 cm for conifers and 30.5 cm for hardwoods, were counted in the four, 2.07 m radius subplots. The minimum size for seedlings is intended to exclude first-year seedlings, which can have a very high mortality rate, and include only well-established individuals. For most species, stem diameter was measured at 1.37 m above the ground. However, for woodland species that frequently have multiple stems (*Pinus monophylla*, *Acer glabrum* and *Cercocarpus ledifolius*), the diameter of all stems was measured at the root collar and the quadratic mean diameter was recorded. Details of the plot design and measurement protocols are available in [34] and [35].

Tree age is difficult to assess and was not measured. Instead, tree size is typically used to define the seedling and mature tree cohorts. Other studies that followed a similar approach defined seedlings and mature trees as individuals with stem diameter smaller or greater than 2.54 cm [23, 24], individuals with diameter smaller 2.54 cm or greater than 12.7 cm [25], or individuals with height less than 50 cm or greater than 8 m [22], respectively. The first criterion does not allow for any temporal separation between the two life stages, so that seedlings and mature trees may actually be coetaneous, while the last two use the same size threshold for all species, regardless of the species’ mature size. In our study, the inventory design determined the definition of seedlings to individuals with stem diameter less than 2.54 cm. However, because tree size and growth rate vary greatly among species, we defined the mature cohort as trees with diameter greater than or equal to the 75th percentile of the estimated species’ diameter distribution in the study area. This threshold diameter ranged between 6.1 and 31.0 cm (median 17.8 cm, Table 1). This criterion should ensure that the mature trees were established well before the seedlings, reflecting recruitment during colder past temperatures.

**Table 1:** List of species included in the study^1^.

		75th percentile diameter cutoff (cm)	Number of plots	
Species	Symbol		Seedlings	Trees
*Abies amabilis*	ABAM	15.2	628	719
*Abies concolor*	ABCO	21.1	1256	1586
*Abies grandis*	ABGR	15.2	883	1044
*Abies lasiocarpa*	ABLA	14.7	450	459
*Abies magnifica*	ABMA	21.1	361	472
**Abies procera**	ABPR	24.4	86	163
*Callitropsis nootkatensis*	CANO4	13.0	94	92
*Calocedrus decurrens*	CADE27	16.8	861	1088
**Chamaecyparis lawsoniana**	CHLA	14.0	28	57
*Juniperus occidentalis*	JUOC	23.4	465	786
*Larix occidentalis*	LAOC	24.4	170	461
*Picea engelmannii*	PIEN	19.6	246	384
*Picea sitchensis*	PISI	28.7	76	168
*Pinus albicaulis*	PIAL	15.5	101	140
*Pinus contorta*	PICO	14.5	813	1403
*Pinus jeffreyi*	PIJE	30.7	178	484
*Pinus lambertiana*	PILA	26.7	437	559
*Pinus monophylla*	PIMO	26.4	111	217
*Pinus monticola*	PIMO3	17.5	275	358
*Pinus ponderosa*	PIPO	23.1	1402	2848
**Pinus sabiniana**	PISA2	31.0	75	171
*Pseudotsuga menziesii*	PSME	25.4	2945	5641
**Sequoia sempervirens**	SESE3	24.4	149	243
*Taxus brevifolia*	TABR2	8.6	186	157
*Thuja plicata*	THPL	14.7	580	989
*Tsuga heterophylla*	TSHE	18.5	1360	1884
*Tsuga mertensiana*	TSME	20.3	380	484
*Acer glabrum*	ACGL	7.6	204	200
*Acer macrophyllum*	ACMA3	17.8	273	746
**Aesculus californica**	AECA	11.4	64	110
*Alnus rubra*	ALRU2	20.3	221	1103
*Arbutus menziesii*	ARME	19.6	330	713
**Chrysolepis chrysophylla**	CHCHC4	11.4	288	261
*Cercocarpus ledifolius*.	CELE3	19.8	121	267
*Cornus nuttallii*	CONU4	6.1	190	126
**Fraxinus latifolia**	FRLA	12.2	40	75
**Lithocarpus densiflorus**	LIDE3	13.2	800	650
*Populus balsamifera*	POBAT	25.9	48	89
*Populus tremuloides*	POTR	8.9	110	93
*Quercus agrifolia*	QUAG	27.9	121	203
*Quercus chrysolepis*.	QUCH2	14.2	1003	923
**Quercus douglasii**	QUDO	22.1	86	417
*Quercus garryana*	QUGA4	15.7	203	362
**Quercus kelloggii**	QUKE	19.8	487	897
*Quercus wislizeni*	QUWI2	11.4	238	291
**Umbellularia californica**	UMCA	11.2	302	311

Bold names indicate that the entire range of the species is within the study region [37, 38]. Individuals with diameter greater than or equal to the 75^th^ percentile diameter cutoff are considered trees.

^1^Native species not included in the study were *Acer negundo*, *Alnus rhombifolia*, *Betula occidentalis*, *Betula papyrifera*, *Hesperocyparis bakeri*, *Hesperocyparis forbesii*, *Hesperocyparis macrocarpa*, *Hesperocyparis sargentii*, *Juniperus californica*, *Juniperus osteosperma*, *Juniperus scopulorum*, *Sequoiadendron giganteum*, *Olneya tesota*, *Prosopis glandulosa*, *Prosopis pubescens*, *Quercus engelmanii*, *Quercus lobata*, *Juglans californica*, *Juglans hindsii*, *Larix lyallii*, *Picea breweriana*, *Pinus attenuata*, *Pinus balfouriana*, *Pinus coulteri*, *Pinus flexilis*, *Pinus longaeva*, *Pinus muricata*, *Pinus radiata*, *Pinus washoensis*, *Pseudotsuga macrocarpa*, *Platanus racemosa*, *Malus fusca*, *Prunus emarginata*, *Prunus virginiana*, *Populus fremontii*, *Torreya californica*.

The sample included 91 tree species, but 9 were non-native and at most occurred in 2 plots. The sample size for some native species was very small, because of their rarity, or because most of their range was outside the study region. Thus, we only included species that were tallied in at least 25 plots as mature trees and 25 plots as seedlings. In addition, *Prunus emarginata* has two varieties with different growth habits: a small tree in the lowlands of western Oregon and Washington (var. *mollis*) and a shrub, typically in higher elevations (var. *emarginata*) [36]. Because the inventory did not discriminate between the two varieties, this species was excluded from the analysis, leaving a total of 46 species (Table 1). The range of 11 species was entirely within the study area [37, 38]. Maps of the distribution of all species, within the study area, are included as supporting information (S1 Fig.).

For each plot, we obtained the mean annual temperature from a spatially gridded (800 m) annual average for the climatological period 1971–2000, developed by the parameter-elevation regressions on independent slopes model (PRISM) [39].

### Statistical Analysis

We followed standard survey sampling procedures [19], albeit from a continuous population perspective [40]. For each species, we computed an approximate design unbiased estimator of the mean elevation, latitude and annual temperature of the range of the seedlings or mature trees, using a weighted domain sample mean. The weights accounted for the different plot density in California and Oregon vs. Washington. We estimated the difference between the mean of the range of seedlings minus that of the range of mature trees as the difference between their respective domain ratio estimators. We estimated approximate variances using a Taylor linearization method and confidence intervals based on the asymptotic normal distribution of the estimators. To estimate changes in the boundary of the species’ temperature range, we compared the 5th and 95th percentiles of the seedling and tree temperature distributions. Estimating the extremes of a distribution requires a larger sample size than estimating the mean. Therefore, we only considered the 36 species present in at least 100 plots as seedlings and 100 plots as mature trees. We used the inverse of the empirical distribution function to estimate the 5th and 95th percentiles of the seedling and mature tree distributions, and the bootstrap to obtain confidence intervals. We estimated overall mean differences, across all species, as the average of the individual species’ differences, weighted by the inverse of the estimated covariance matrix. This approach accounts for both the lack of independence among the individual species’ estimators, because they are derived from the same set of plots, and the wide range of their variances, in part due to large differences in realized sample sizes (for details of the statistical analysis, see S1 Appendix).

## Results and Discussion

**Fig 2:**
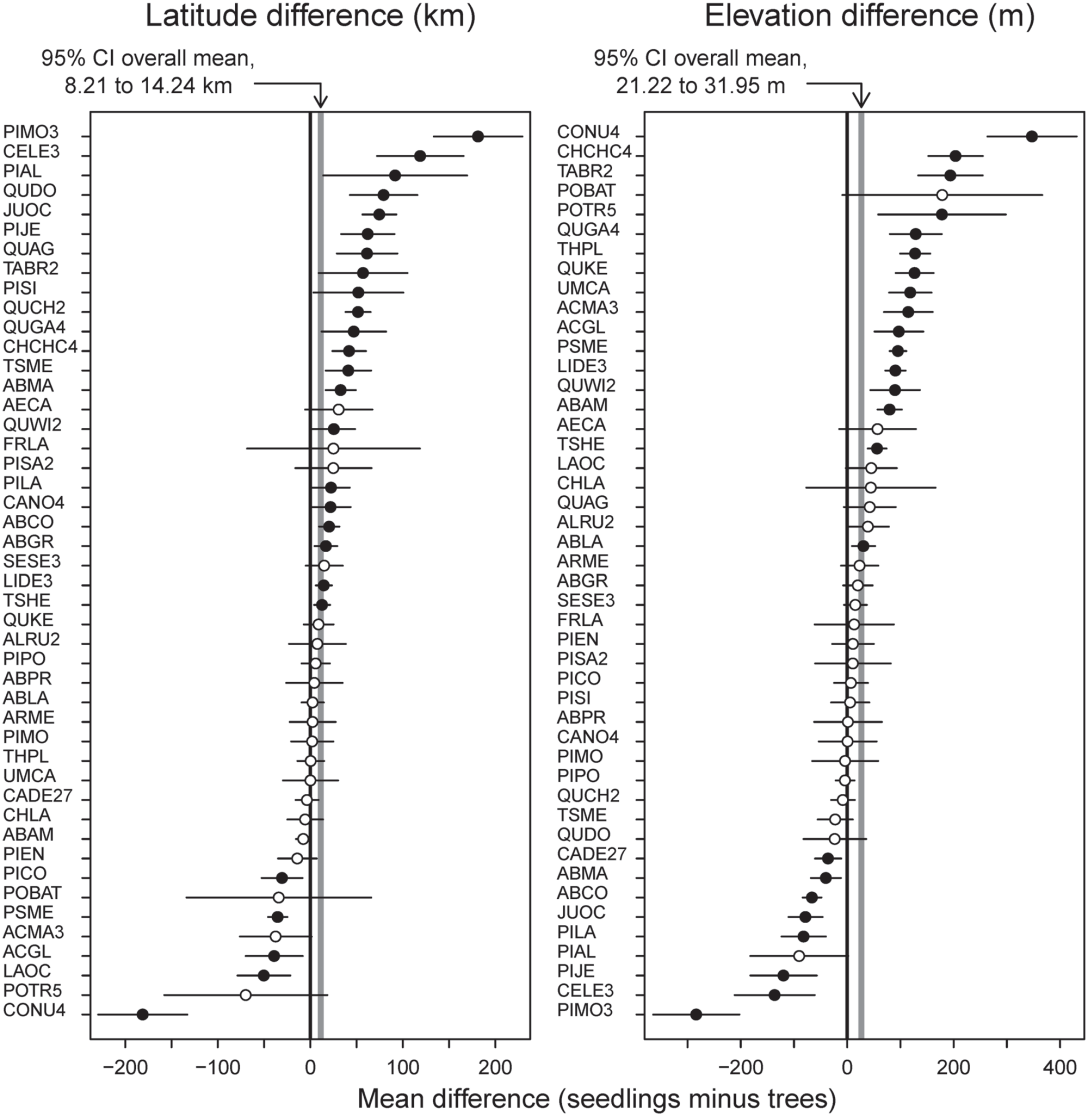
Difference between the mean latitude and elevation of the range of seedlings and mature trees. A positive number indicates that the mean of the seedling range is higher than or north of that of trees. The circles represent the estimated difference for each species and the horizontal lines represent 95% confidence intervals for the difference. Solid circles indicate that the 95% CI does not include 0 (difference significant at the 0.05 level), open circles indicate that the 95% CI includes 0. The gray band is a 95% confidence interval for the overall mean difference, across all species. Species name codes listed in Table 1.

**Fig 3:**
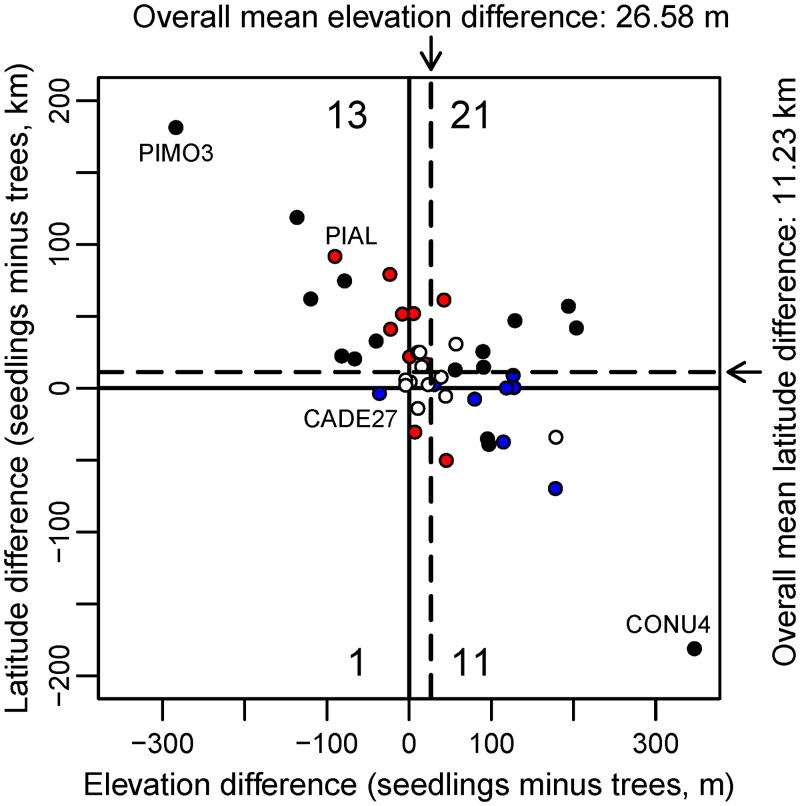
Latitudinal and elevational range shifts. A positive number indicates that the mean of the seedling range is higher than or to the north of that of trees. Dashed lines represent the overall mean difference across all species. White circles represent species for which neither the difference in elevation or latitude is significantly different from 0 at the 0.05 level; red circles represent species for which the difference in latitude is different from 0, but not that of elevation; blue circles represent species for which the difference in elevation is different from 0, but not that of latitude; black circles represent species for which the difference in both latitude and elevation is different from 0. The numbers in each quadrant represent the number of species in that quadrant. Species mentioned in the text are labeled: *Pinus monticola* (PIMO3), *Pinus albicaulis* (PIAL), *Calocedrus decurrens* (CADE27), *Cornus nuttallii* (CONU4).

The mean elevation and latitude of the range of seedlings was higher than or north of the mean of the range of mature trees for most species (32 and 34 out of 46 species, respectively, of which 21 and 16 were different from 0 at the 0.05 level, Fig. 2). Averaged across all species, the mean of the distribution of seedlings was 26.58 m (95% C.I. from 21.22 to 31.95 m) higher than and 11.22 km (95% C.I. from 8.21 to 14.24 km) north of that of mature trees. When changes in elevation and latitude were examined jointly (Fig. 3), the mean of the seedling distribution of 21 species was both higher than and north of that of mature trees, a response consistent with a warming climate. Seedlings of only one species, *Calocedrus decurrens*, showed the opposite trend, i.e., a decrease in elevation and a southward shift in latitude, although the differences were small. However, for the remaining 24 species, the mean elevation and the mean latitude of the seedling range was either higher and more southern, or vice versa. The most extreme cases, *Pinus monticola* and *Cornus nuttallii*, show very large, opposing differences: seedlings for the former were 181 km north and 283 m lower than mature trees and, for the latter, 181 km south and 347 m higher (Fig. 3). For the 24 species that show opposing latitudinal and elevational trends, examining latitude or elevation change separately would lead to contradictory conclusions regarding their response to climate warming.

**Fig 4:**
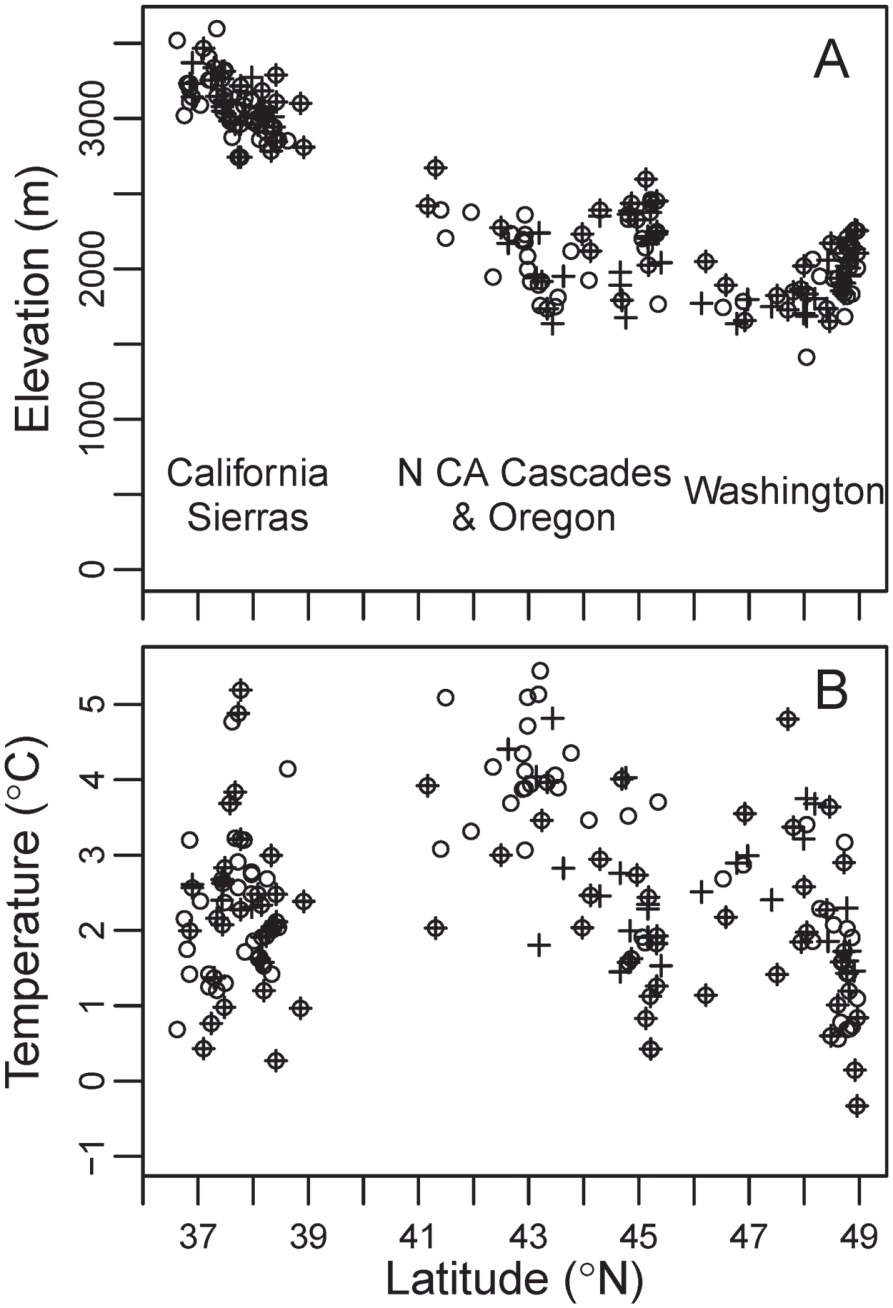
Distribution of plots with *Pinus albicaulis*. Open circles represent plots with trees, and crosses, plots with seedlings. (A) Elevation and (B) mean annual temperature, as a function of plot latitude.

The apparent contradiction in the elevation and latitude response is likely the result of confounding between those two variables. For example, *Pinus albicaulis*, a high elevation, timberline pine, shows large, opposite changes in elevation and latitude. Across the region, the mean elevation of the range of seedlings is 90.0 m lower than that of the range of mature trees, while the mean latitude is 91.8 km north (Fig. 3). However, the elevation of the species’ range decreases as the latitude increases: the mean elevation is 3,090 m in the California Sierras population (mean latitude 37.75° N) and 1,920 m in the Washington populations (mean latitude 48.19° N), while the mean annual temperature of the two populations remains similar (2.26 vs. 2.00°C, respectively) (Fig. 4). The greater seedling frequency in the northern population suggests that the species distribution is shifting northwards: the ratio of the number of plots with seedlings to plots with mature trees is 0.87 in Washington and 0.59 in the California Sierras. However, as the population distribution shifts northwards, its mean elevation also decreases.

**Fig 5:**
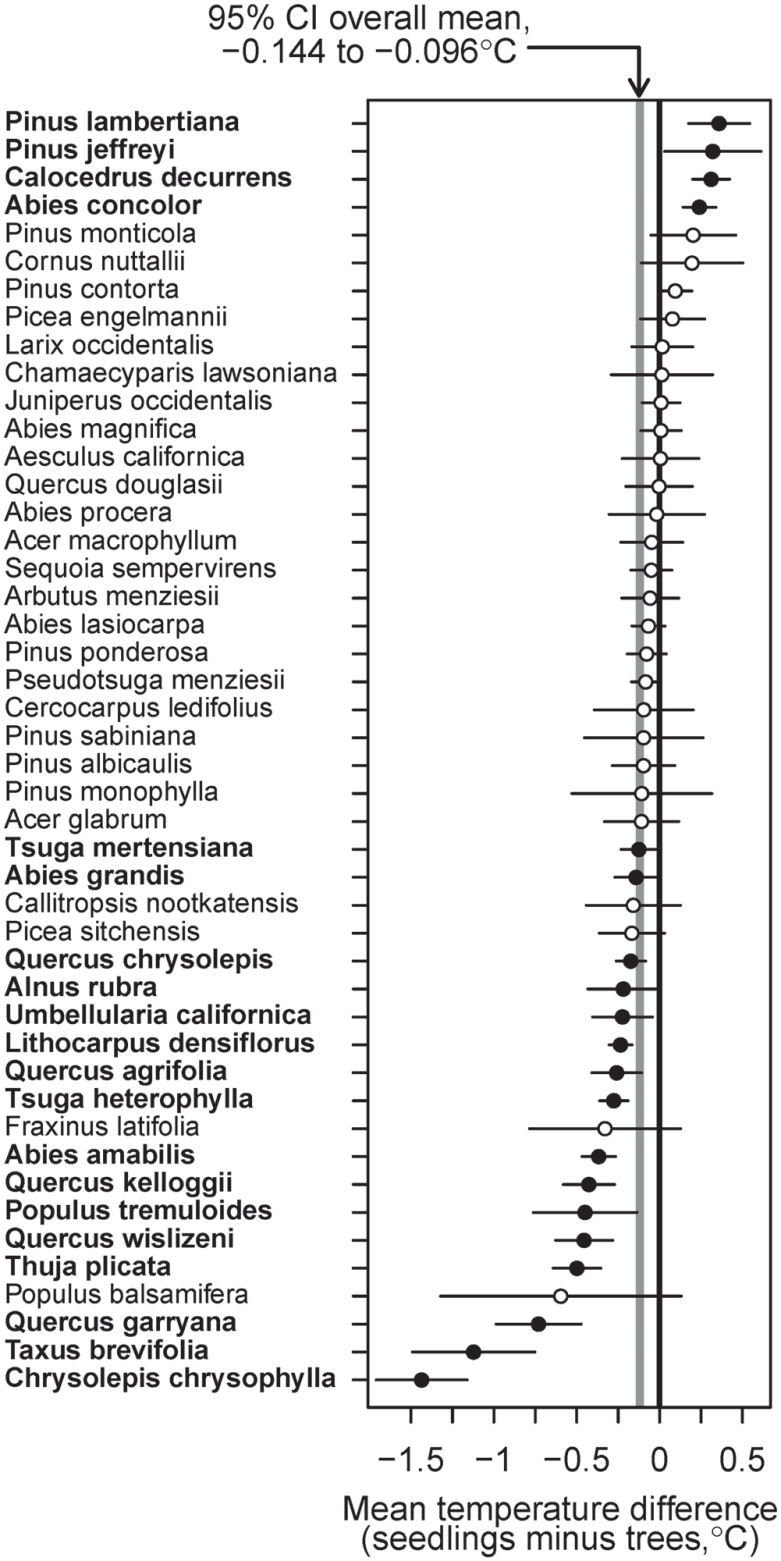
Difference between the mean temperature of the range of seedlings and that of mature trees. A positive number indicates that the mean temperature of the seedling range is warmer than that of trees. The circles represent the estimated difference for each species and the horizontal lines represent 95% confidence intervals for the difference. Solid circles and bold species’ name indicate that the 95% C.I. does not include 0 (difference significant at the 0.05 level). The gray band is a 95% C.I. for the overall mean difference, across all species.

Global warming, and thus increased temperature, is assumed to be the direct cause of the poleward or upward shifts in species distributions. Therefore, rather than examine changes in surrogates that may be confounded, such as elevation or latitude, the hypothesis can be assessed directly by estimating whether the seedling distribution has shifted towards areas of colder temperature, relative to that of mature trees. Across all species, the mean annual temperature of the range of seedlings was 0.120°C lower than that of the range of trees (95% C.I. 0.096 to 0.144°C), consistent with a range shift caused by global warming. For 33 (16 statistically significant at the 0.05 level) out of 46 species, the mean annual temperature of the range of seedlings was colder than that of the range of trees (Fig. 5). The magnitude of the difference was very small for the majority of the 13 species that showed the opposite trend. A shift towards warmer areas was statistically significant (0.05 level) only for 4 species: *Pinus lambertiana*, *Pinus jeffreyi*, *Calocedrus decurrens* and *Abies concolor*, major components of the California mixed conifer forests. This forest type has shown widespread mature tree mortality, concentrated at the lower or drier end of its range, without a marked decrease in seedling recruitment [7, 29, 41].

We used the mean of the temperature distribution to assess shifts on the species’ ranges because it incorporates information from the entire distribution, including the minimum and maximum; is commonly used in species distribution and climate prediction models; and is highly correlated with other temperature variables. However, the maximum and minimum temperature can be very important determining species range. The conclusions of the study did not change when using those metrics: across all species, the minimum and maximum annual temperatures of the range of seedlings were 0.143°C (95% C.I. -0.119 to -0.169°C) and 0.100°C (95% C.I. -0.072 to -0.129°C) lower than those of the range of trees. These results suggest that there is tendency for the range to shift towards areas where the winters are relatively colder than the summers, consistent with a shift towards the interior areas, with a more pronounced continental climate.

**Fig 6:**
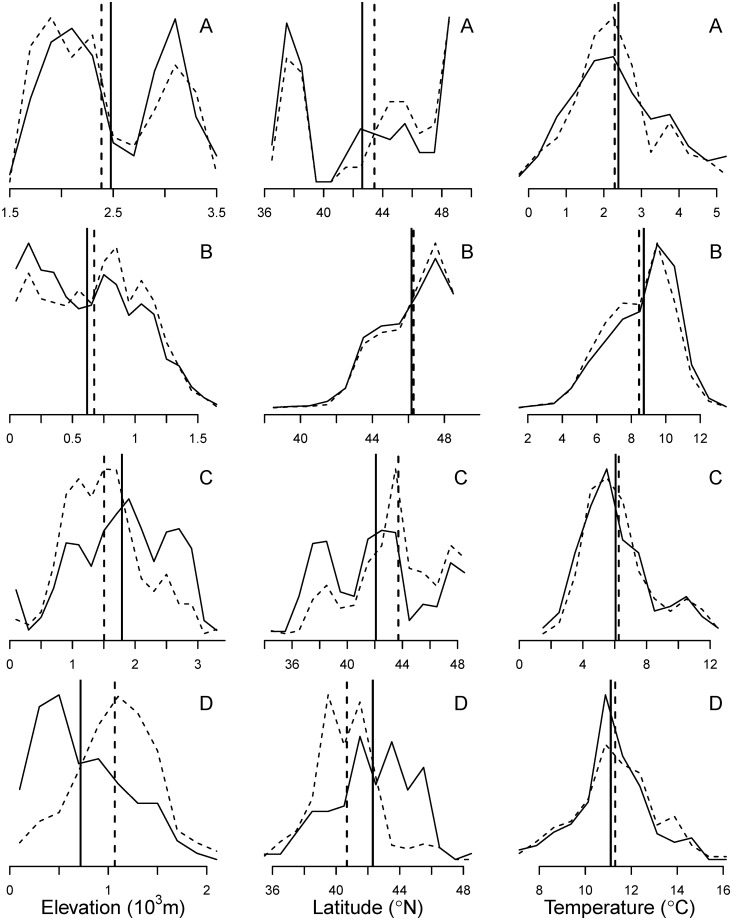
Estimated probability density functions of the elevation, latitude and temperature of the range of selected species. (A) *Pinus albicaulis*; (B) *Tsuga heterophylla*; (C) *Pinus monticola*; (D) *Cornus nuttallii*. The solid line represents the distribution of mature trees and the dashed line the distribution of seedlings. Vertical lines represent the estimated mean of the distribution. The first column shows the elevation, the second latitude, and the third mean annual temperature. Ordinates are not shown because the figures are scaled so that the area under the curves is 1.

There are additional advantages of examining species’ shifts in temperature, rather than in elevation or latitude. Species’ elevation and latitudinal distributions are highly dependent on the peculiarities of geography, which may also condition the apparent response to climate warming. In contrast, the shape of the temperature distribution tends to be better behaved and less affected by idiosyncrasies of the study region (Fig. 6). For example, the elevation and latitude distributions of *Pinus albicaulis* are multimodal, matching the distribution of the highest mountain ranges, with the latter truncated at the limit of the study area, the Canadian border (Fig. 6A). The species is largely absent between the California Sierras and the northern California and southern Oregon Cascades, because this region that lacks mountain ranges of sufficiently high elevation. For many species, the elevation distribution is truncated at sea level, where the mode may be located, or the latitudinal distribution at the study area boundaries or geographic barriers (e.g., *Tsuga heterophylla*, Fig. 6B). A species’ geographic distribution may also reflect complex interactions with other factors such as pathogens, rather than response to climate change. The ranges of *Pinus monticola* and *Cornus nuttallii*, the two species with the greatest discrepancy in the latitude and elevation response (Fig. 2), have been significantly affected by introduced pathogens (white pine blister rust, *Cronartium ribicola* [42], and dogwood anthracnose, *Discula destructiva*, respectively). The distribution of *Pinus monticola* is multimodal and very complex, probably driven by the interaction between geographic factors and the impact of blister rust (Fig. 6C). Dogwood anthracnose is a relatively recent introduction and not much is known about its effects in the western US. However, studies in a similar tree species indicate high mortality, greatest among smaller size classes and in moister sites [43]. This pattern could explain the relative greater frequency of mature trees in the northern and lower areas of the range, corresponding to the populations of moist western Oregon and Washington, and the relatively greater abundance of seedlings south and higher, in the comparatively dry California Sierras (Fig. 6D). For those two species, however, the temperature distributions of the range of seedlings and mature trees are very similar, despite the large differences in the elevation and latitude distributions, suggesting that the elevation and altitude differences are caused by factors other than temperature change.

**Fig 7:**
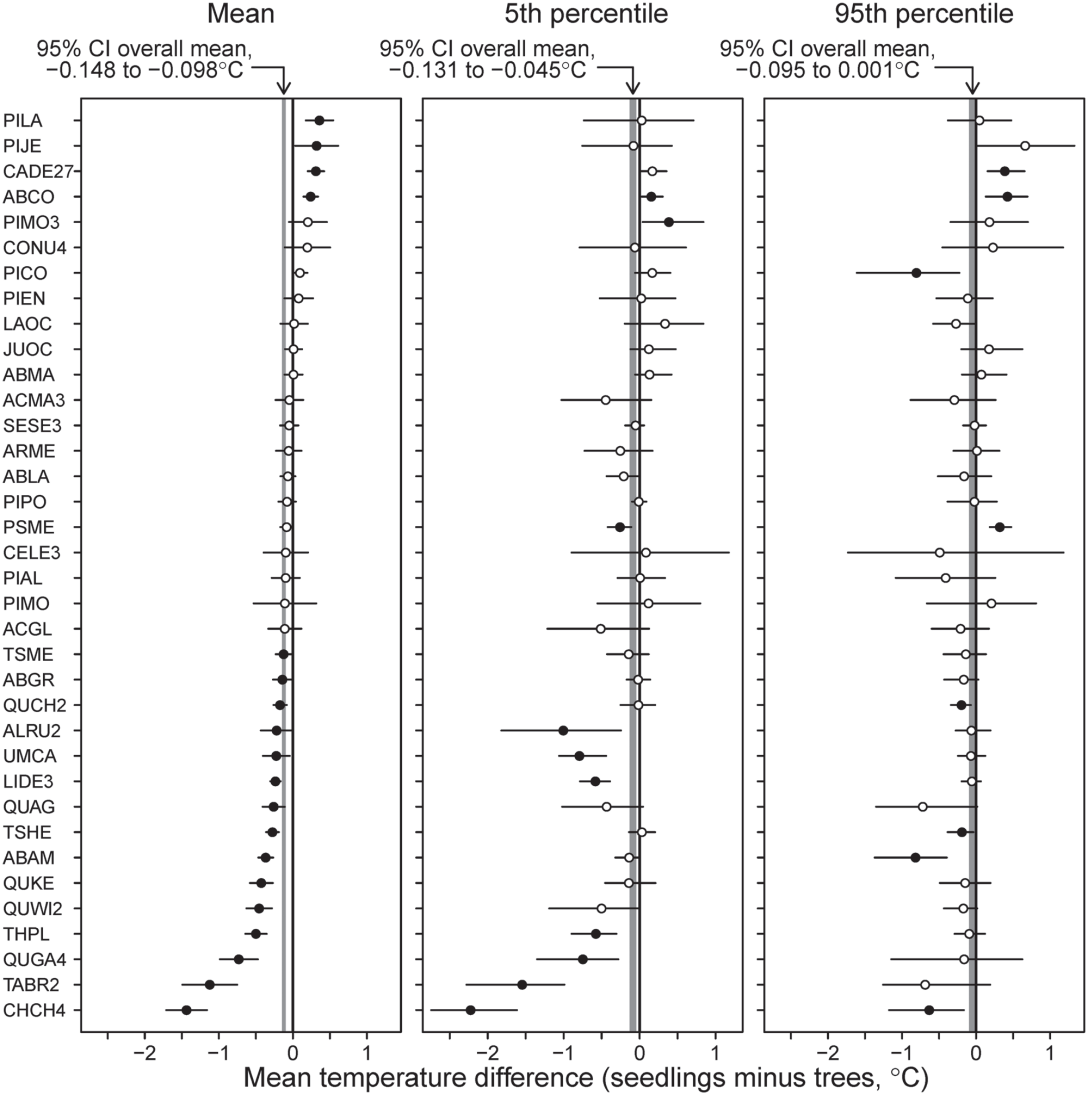
Difference between the mean and percentiles of the temperature distribution of seedlings and that of mature trees. A positive number indicates that the mean temperature of the seedling range is warmer than that of trees. The circles represent the estimated difference for each species and the horizontal lines represent 95% confidence intervals for the difference. Solid circles indicate that the 95% CI does not include 0 (difference significant at the 0.05 level), open circles indicate that the 95% CI includes 0. The gray band is a 95% confidence interval for the overall mean difference, across all species. Only species present at more than 100 plots as seedlings and as trees are included. Species name codes listed in Table 1.

Averaged across the 36 species present in at least 100 plots, the 95th and 5th percentiles of the temperature distribution of seedlings were 0.047°C (95% C.I. -0.001 to 0.095°C) and 0.088°C (95% C.I. 0.045 to 0.131) colder than those of the distribution of mature trees (Fig. 7). The mean temperature difference estimated from those 36 species was almost identical to that estimated with the full set of 46 species: 0.123 vs. 0.120°C, respectively. While the change in both percentiles averaged across all species indicates a shift of entire distributions towards cooler areas, the magnitude of the difference of the range limits was less pronounced than that of the central tendency. The results are in accordance with studies in the Eastern United States, which found a lack of northward shifts in the boundary of individual species’ latitudinal range [24]. Our results suggest a change in the shape of the frequency distribution, so that the range footprint remains relatively stable but species’ relative abundance within the range increases towards the colder end of their range [27, 44]. The warm end of the range changed the least, but tree longevity and the ability of established trees to withstand adverse environmental conditions may delay local extinction, even if the population is not sustainable [28, 45]. The presence of mature trees could provide a source of seedlings which may survive at least during periods of favorable weather. On the colder end of the range, the long time required by many trees to reach reproductive maturity may slow species’ expansion.

A large difference in temperature response was noted between the two main taxonomic groups: for angiosperms, the mean temperature of the range of seedlings was 0.246°C lower than that of the range of trees (95% C.I. 0.207 to 0.292°C), while for gymnosperms it was only 0.059°C (95% C.I. 0.030 to 0.089°C). The two groups differ in many ecological traits, including dispersal mechanisms, seed size and longevity, and in their geographical distribution. Angiosperms are more common in the southern, warmer and dryer end of the region, or restricted to special habitats, such as riparian forests. The different responses of angiosperms and gymnosperms are not easily explained and warrant further study.

The results of this study rely on the assumption that tree size is a surrogate for tree age. To assess the sensitivity of the results to the definition of mature tree, we repeated the analysis using the 60th and 85th percentiles of the tree diameter distribution as the threshold diameter. The median diameter of the 46 species changed from 18.2 cm when using the 75th percentile, to 11.4 and 24.9 cm for the 60th and 85th percentiles, respectively. The results, however, were very stable: the mean temperature of the range of seedlings was 0.124 and 0.121°C colder than that of trees for the 60th and 85th percentiles, respectively. A potentially more important effect is the link between small tree size and younger age. Most angiosperms, for example, tend to have a flexible growth habit and may sprout after disturbance or when stressed. Sprouting or developing a shrubby growth form could result in tallying the individual as a seedling, instead of a mature tree. Individuals at the edge of the species distribution may grow more slowly, thus spending more time as seedlings. However, there is no indication of a bias that would result from those processes affecting the colder, but not the warmer, end of the range.

## Conclusions

Estimating differences in elevation or latitude to establish the impact of climate change may lead to spurious results, due to confounding effects and artifacts caused by geographic peculiarities of the study region. The majority of the tree species included in this study showed opposing elevational and latitudinal shifts which, if examined separately, would result in contradictory and possibly incorrect conclusions about their response to a warming climate. Spatially referenced climate observations and models allow estimation of shifts in the species’ distributions along temperature gradients, a variable that has a direct causal interpretation in the context of climate change. Compared with the artifacts that afflict analysis of elevation and latitude, the influence of small errors in the temperature models is likely to be minor for broad-scale assessments with a large, spatially balanced sample.

Temperature change is one of the many factors that can drive shifts in tree species distributions in a complex landscape. Changes in disturbance regimen, land use, geographic idiosyncrasies, and other variables may affect range changes, masking the strength, and even direction, of the response to a warming climate. European settlement was relatively recent in the study region, resulting in large impacts in forested lands and large-scale changes of species distributions through fire suppression, the introduction of grazing and exotic pathogens, intensive forest management, and the expansion of agriculture and urbanization. However, the results of this study show that, across all species and despite individual species’ idiosyncratic responses, there is a significant shift of the distribution of seedlings towards colder environments, relative to the distribution of mature trees. The large geographic scale and environmental diversity of our study area, the large number and exhaustive sampling of tree species, and the direct causal relationship between the response and the hypothesized cause, provide strong evidence to attribute those shifts to climate change.

## Supporting Information

S1 Fig Species range maps. Maps of the plots containing the species included in the study. Species name codes listed in Table 1
(PDF)

S1 Appendix Details of the statistical analysis. (PDF)

## References

[1] Chen IC, Hill JK, Ohlemüller R, Roy DB, Thomas CD (2011) Rapid range shifts of species associated with high levels of climate warming. Science 333: 1024–1026. 10.1126/science.1206432 21852500

[2] Kerr JT, Kharouba HM, Currie DJ (2007) The macroecological contribution to global change solutions. Science 316: 1581–1584. 17569854 10.1126/science.1133267

[3] Parmesan C, Duarte C, Poloczanska E, Richardson AJ, Singer MC (2011) Overstretching attribution. Nat Clim Chan 1: 2–4.

[4] Hawkins BA, Diniz-Filho JAF (2004) ‘Latitude’ and geographic patterns in species richness. Ecography 27: 268–272.

[5] Feeley KJ, Silman MR, Bush MB, Farfan W, Garcia Cabrera K, et al (2011) Upslope migration of Andean trees. J Biogeogr 38: 783–781.

[6] Kelly AE, Goulden ML (2008) Rapid shifts in pant distribution with recent climate change. Proc Natl Acad Sci USA 105: 11823–11826. 10.1073/pnas.0802891105 18697941 PMC2575286

[7] Fellows AW, Goulden ML (2012) Rapid vegetation redistribution in Southern California during the early 2000s drought. J Geophys Res 117, G03025.

[8] Schwilk DW, Keeley JE (2011) A plant distribution shift: temperature, drought or past disturbance? PLoS One 7, e31173. 10.1371/journal.pone.0031173 PMC3277505 22348051

[9] Lenoir J, Gégout JC, Marquet PA, de Ruffray P, Brisse H (2008) A significant upward shift in plant species optimum elevation during the 20th century. Science 320: 1768–1771. 10.1126/science.1156831 18583610

[10] Crimmins SM, Dobrowski SZ, Greenberg JA, Abatzoglou JT, Mynsberge AR (2011) Changes in climatic water balance drive downhill shifts in plant species’ optimum elevation. Science 331: 324–327. 10.1126/science.1199040 21252344

[11] Hill JK, Thomas CD, Fox R, Telfer MG, Willis SG, et al (2002) Responses of butterflies to twentieth century climate warning: implications for future ranges. Proc R Soc Lond B Biol Sci 269: 2163–2171. 10.1098/rspb.2002.2134 PMC1691143 12396492

[12] Heckman JJ (1979) Sample selection bias as a specification error. Econometrica 47: 153–161.

[13] Wolf A, Anderegg WRL (2011) Comment on “Changes in climatic water balance drive downhill shifts in plant species’ optimum elevations”. Science 334: 177 10.1126/science.1205740 21998369

[14] Hijmans RJ (2011) Comment on “Changes in climatic water balance drive downhill shifts in plant species’ optimum elevations”. Science 334: 177 10.1126/science.1205740 21998370

[15] Stephenson NL, Das AJ (2011) Comment on “Changes in climatic water balance drive downhill shifts in plant species’ optimum elevations”. Science 334: 177 10.1126/science.1205740 21998371

[16] Holzinger B, Hülber K, Camenisch M, Grabherr G (2008) Changes in plant species richness over the last century in the eastern Swiss Alps: elevational gradient, bedrock effects and migrations rates. Plan Ecol 195: 179–196.

[17] Bertrand R, Lenoir J, Piedallu C, Riofrio-Dillon G, de Ruffray P, et al (2011) Changes in plant community composition lag behind climate warming in lowland forests. Nature 479: 517–520. 10.1038/nature10548 22012261

[18] Warren MS, Hill JK, Thomas JA, Asher J, Fox R, et al (2001) Rapid response of British butterflies to opposing forces of climate and habitat change. Nature 414: 65–69. 11689943 10.1038/35102054

[19] Särndal CE, Swensson B, Wretman J (1992) Model Assisted Survey Sampling. New York: Springer-Verlag 694 p.

[20] Grubb PJ (1977) The maintenance of species-richness in plant communities: the importance of the regeneration niche. Biol Rev 52: 107–145.

[21] Horn HS (1975) Markovian processes of forest succession In: Cody ML, Diamond JM, editors. Ecology and evolution of communities. Cambridge: Belknap pp.196–211.

[22] Lenoir J, Gégout JC, Pierrat JC, Bontemps JD, Dhôte JF (2009) Difference between tree species seedling and adult altitudinal distribution in mountain forests during the recent warm period (1986–2006). Ecography 32: 765–777.

[23] Woodall CW, Oswalt CM, Westfall JA, Perry CH, Nelson MD, et al (2009) An indicator of tree migration in forests of the eastern United States. For Ecol Manage 257: 1434–1444.

[24] Zhu K, Woodall CW, Clark JS (2012) Failure to migrate: lack of tree range expansion in response to climate change. Glob Chan Biol 18: 1042–1052.

[25] Bell DM, Bradford JB, Laurenroth WK (2014) Early indicators of change: divergent climate envelopes between tree life stages imply range shifts in the western United States. Global Ecol Biogeogr 23: 168–180.

[26] Zhu K, Woodall CW, Ghosh S, Gelfand AE, Clark JS (2014) Dual impacts of climate change: forest migration and turnover through life history. Glob Chan Biol 20: 251–264. 10.1111/gcb.12382 24014498

[27] Murphy HT, VanDerWal J, Lovett-Doust J (2010) Signatures of range expansion and erosion in eastern North American trees. Ecol Lett 12: 1233–1244. 10.1111/j.1461-0248.2010.01526.x 20735463

[28] Jump AS, Mátyás C, Peñuelas J (2009) The altitude-for-latitude disparity in the range retractions of woody species. Trends Ecol Evol 24: 694–701. 10.1016/j.tree.2009.06.007 19695735

[29] Allen CD, Macalady AK, Chenchouni H, Bachelet D, McDowell N, et al (2010) A global overview of drought and heat induced tree mortality reveals emerging climate risk for forests. For Ecol Manage 259: 660–684.

[30] Smith WB, Miles PD, Perry CH, Pugh SA (2009) Forest Resources of the United Stated, 2007 Gen. Tech. Rep. WO-78, Washington, D.C.: US Forest Service 336 p.

[31] FAO (2000) FRA 2000: On definitions of forest and forest change Forest Resource Assessment Working Paper 33, Rome: UN Food and Agriculture Organization 14 p.

[32] McRoberts RE (2005) The enhanced Forest Inventory and Analysis program In: Bechtold WA, Patterson PL, editors. The enhanced Forest Inventory and Analysis Program—national sampling design and estimation procedures. Gen. Tech. Rep. SRS-80, Asheville: US Forest Service pp. 1–10.

[33] Omernik JM (1987) Ecoregions of the conterminous United States. Ann Assoc Am Geogr 77: 374–378.

[34] Bechtold WA, Scott CT (2005) The Forest Inventory and Analysis plot design In: Bechtold WA, Patterson PL, editors. The enhanced Forest Inventory and Analysis Program—national sampling design and estimation procedures. Gen. Tech. Rep. SRS-80, Asheville: US Forest Service, pp. 27–42.

[35] USDA Forest Service (2008) Field instructions for the annual inventory of California, Oregon and Washington. Available: http://www.fs.fed.us/pnw/rma/fia-topics/documentation/field-manuals/. Accessed December 1, 20124.

[36] Hitchcock CL, Cronquist A (1973) Flora of the Pacific Northwest. Seattle: University of Washington 750 p.

[37] Little EL Jr (1971) Atlas of United States trees, volume 1, conifers and important hardwoods: U.S. Department of Agriculture Miscellaneous Publication 1146, 9 p., 200 maps, available at http://esp.cr.usgs.gov/data/little/. Accessed 2014 Dec 1.

[38] Little EL Jr (1976) Atlas of United States trees, volume 3, minor Western hardwoods: U.S. Department of Agriculture Miscellaneous Publication 1314, 13 p., 290 maps, available at http://esp.cr.usgs.gov/data/little/. Accessed 2014 Dec 1.

[39] Daly C, Halbleib M, Smith JI, Gibson WP, Doggett MK, et al (2008) Physiographically sensitive mapping of climatological temperature and precipitation across the conterminous United States. International Journal of Climatology 28: 2031–2064.

[40] Cordy C (1993) An extension of the Horvitz-Thomson theorem to point sampling from a continuous population. Stat Probab Lett 18: 353–362.

[41] Van Mantgem PJ, Stephenson NL (2007) Apparent climatically induced increase of tree mortality rates in a temperate forest. Ecol Lett 10: 909–916. 17845291 10.1111/j.1461-0248.2007.01080.x

[42] Geils BW, Hummer KE, Hunt RS (2010) White pines, *Ribes*, and blister rust: a review and a synthesis. Forest Pathology 40: 147–185.

[43] Jenkins MA, White PS (2002) *Cornus florida* L. mortality and understory composition changes in western Great Smoky Mountains National Park. Journal of the Torrey Botanical Society 123: 194–206.

[44] Breshears DD, Huxman TE, Adams HD, Zou CB, Davison JE (2008) Vegetation synchronously leans upslope as climate warns. Proc Natl Acad Sci USA 105: 11591–11592. 10.1073/pnas.0806579105 18697950 PMC2575300

[45] Davis MB (1989) Lags in vegetation response to greenhouse warming. Clim Change 15: 75–82. 2725831

