# Supplementary material for: Evidence of Tree Species’ Range Shifts in a Complex Landscape
Source: PLoS One. 2015 Jan 29;10(1):e0118069. doi: 10.1371/journal.pone.0118069 (PMC4310600; doi:10.1371/journal.pone.0118069)
Supplement: S1 Fig — Maps of the plots containing the species included in the study. Species name codes listed in Table 1 (PDF) [file pone.0118069.s001.pdf]

S1 Figure. Species range maps. Maps of the plots containing the species included in the study. Species name codes listed in Table 1.

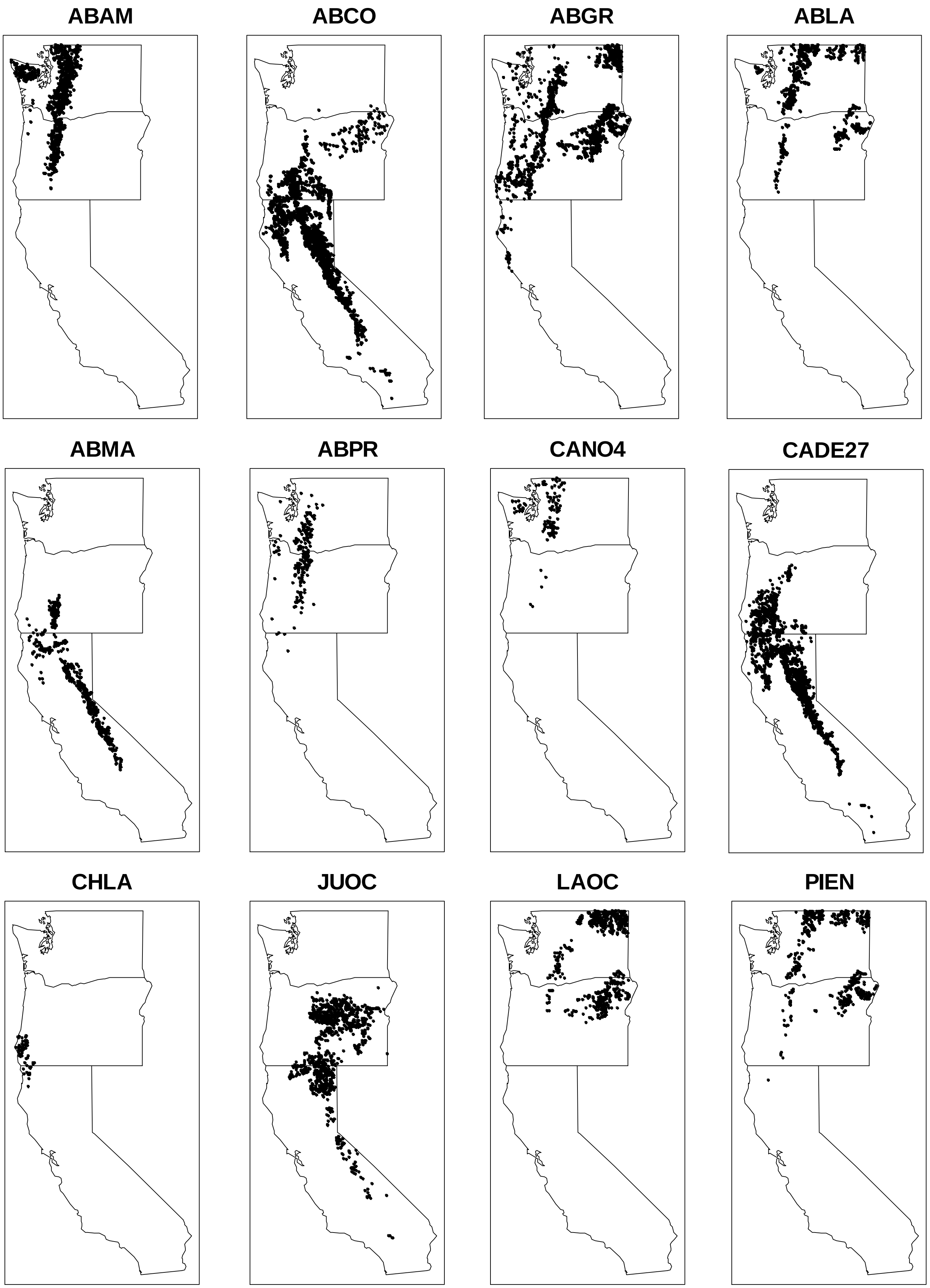
ABAM
ABCO
ABGR
ABLA
ABMA
ABPR
CANO4
CADE27
CHLA
JUOC
LAOC
PIEN

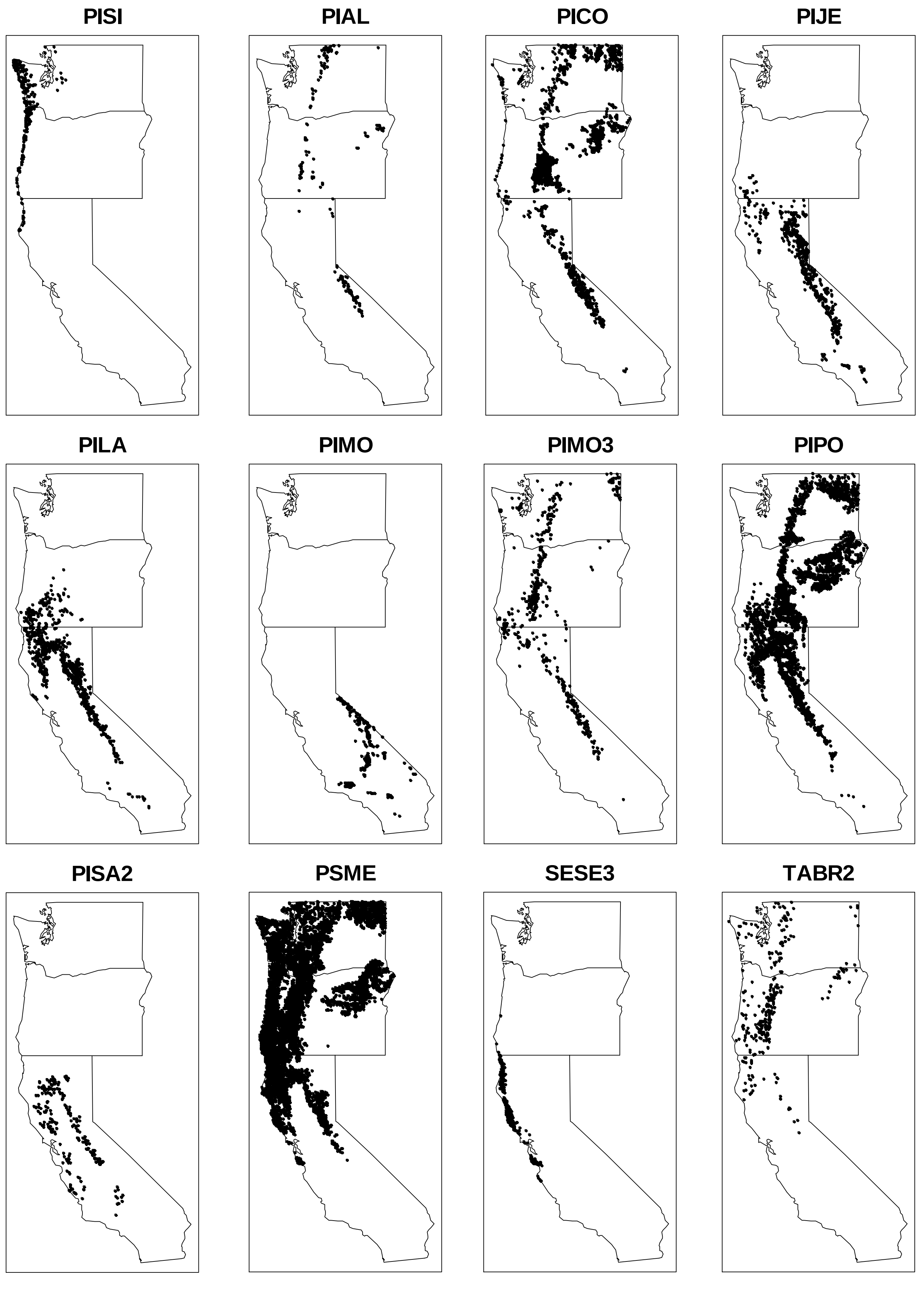
PISI
PIAL
PICO
PIJE
PILA
PIMO
PIMO3
PIPO
PISA2
PSME
SESE3
TABR2

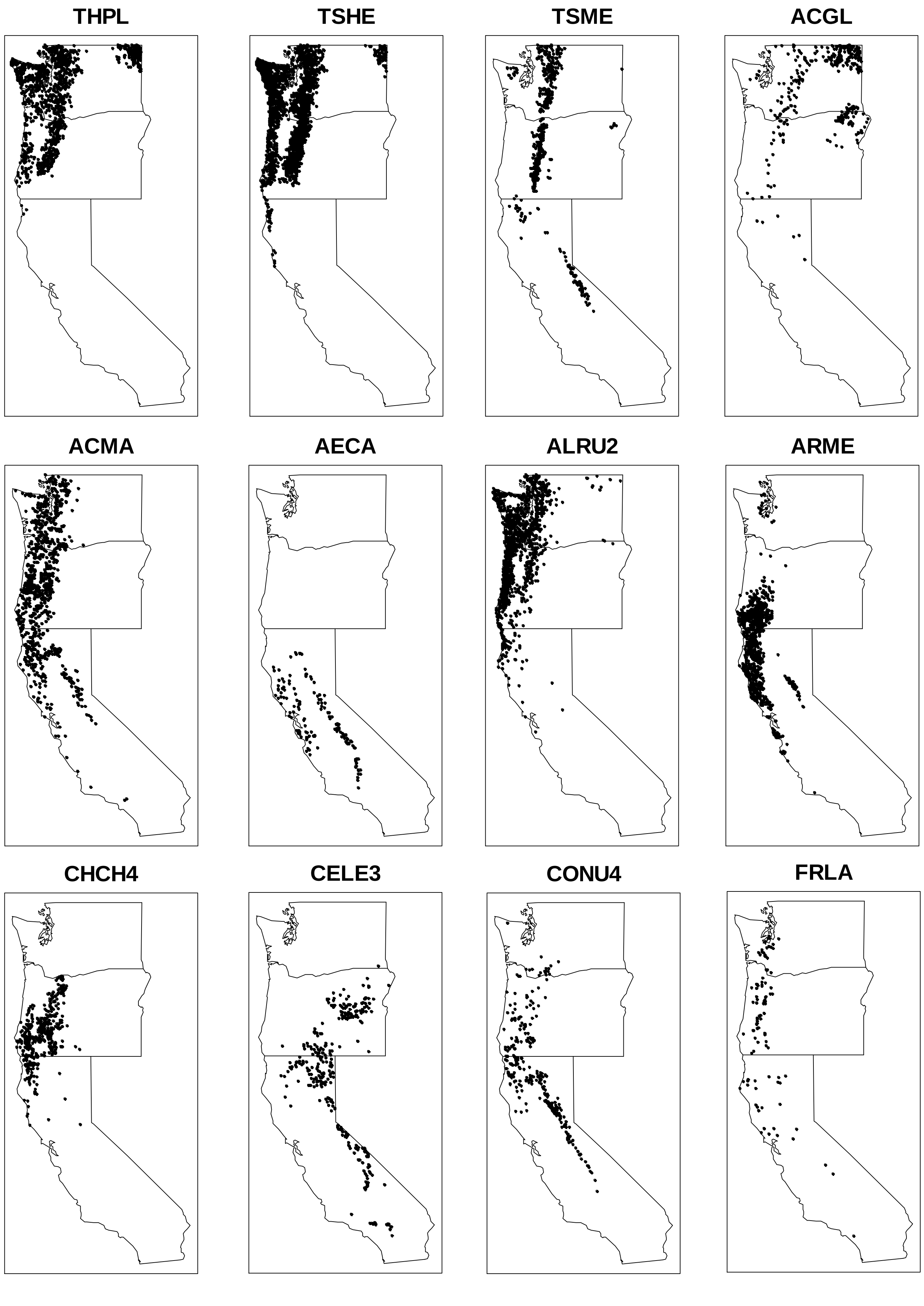
THPL
TSHE
TSME
ACGL
ACMA
AECA
ALRU2
ARME
CHCH4
CELE3
CONU4
FRLA

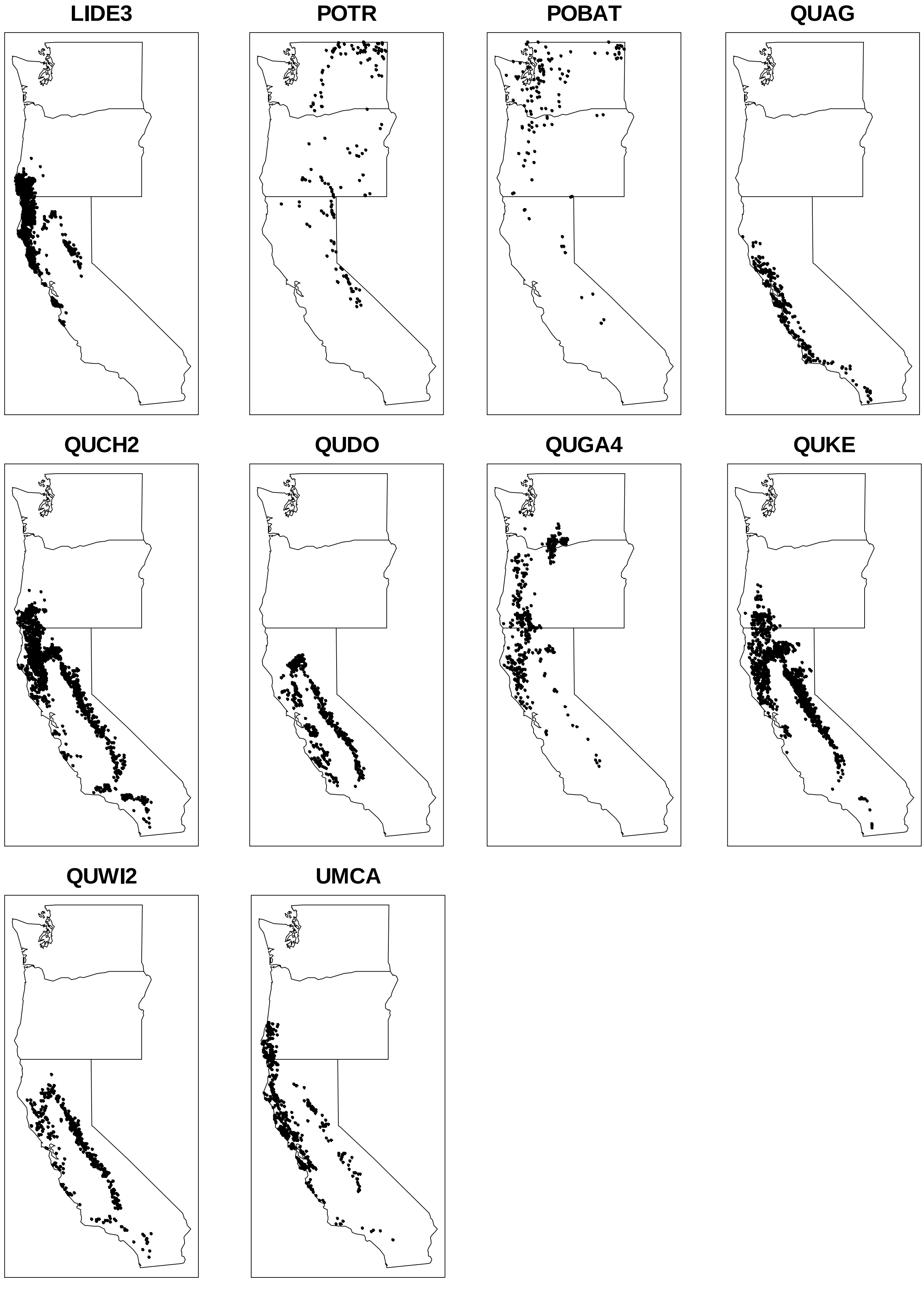
LIDE3
POTR
POBAT
QUAG
QUCH2
QUDO
QUGA4
QUKE
QUWI2
UMCA
