# Supplementary material for: Evidence of Tree Species’ Range Shifts in a Complex Landscape
Source: PLoS One. 2015 Jan 29;10(1):e0118069. doi: 10.1371/journal.pone.0118069 (PMC4310600; doi:10.1371/journal.pone.0118069)
Supplement: S1 Appendix — (PDF) [file pone.0118069.s002.pdf]

S2 Appendix. Details of the statistical analysis

Statistical analysis follows standard survey sampling procedures [1], but from a continuous population perspective [2]. For each species, an approximate design unbiased estimator of the mean elevation, latitude, and annual temperature of the range of seedlings or mature trees ($\hat{\mu}_{kd}$) is given by the weighted domain sample mean (after [1], section 5.8):

$$\hat{\mu}_{kd} = \frac{1}{\left|\hat{D}_{kd}\right|} \sum_{i=1}^{n} I_{kd}\left(p_i\right) y\left(p_i\right) / \pi\left(p_i\right) \qquad (1)$$

$$\left|\hat{D}_{kd}\right| = \sum_{i=1}^{n} I_{kd}\left(p_i\right) / \pi\left(p_i\right) \qquad (2)$$

where k indexes species and d indexes whether the estimator is for mature trees (T) or seedlings (S); $I_{kd}\left(p_i\right)$ is an indicator variable that takes the value of 1 if the i-th plot contains trees or seedlings of the k-th species or 0 otherwise; $y\left(p_i\right)$ and $\pi\left(p_i\right)$ are the value of the variable of interest (latitude, elevation or temperature) and the inclusion density function in the i-th plot, respectively; $\left|\hat{D}_{kd}\right|$ is an estimator of the size of the domain (range of seedlings or trees of the species of interest); and the sum is over n, the total sample size.

The inclusion density function is different for plots in California or Oregon vs. Washington. The sample is divided into 10 spatially balanced panels, so that one panel is measured every year. In California and Oregon, the full 10 panels have been completed (2001-2010), yielding one plot every 24.0 km^2 approximately. In Washington, however, only 9 panels are currently available (2002-2010), yielding one plot every 26.6 km^2 approximately. The inclusion density function $\pi\left(p_i\right)$ is derived by first calculating the selection probability density of a single plot selection, $f\left(p_i\right)$ ([3], section 4.3):

$$f\left(p_i\right)=\begin{cases}10\big/\left[10\left(A_{CA}+A_{OR}\right)+9A_{WA}\right] \text{ if plot } \mathrm{p_i} \text{ is in California or Oregon}\\ 9\big/\left[10\left(A_{CA}+A_{OR}\right)+9A_{WA}\right] \text{ if plot } \mathrm{p_i} \text{ is in Washington}\end{cases} \tag{3}$$

where A_{CA}, A_{OR}, and A_{WA} are the land areas of California, Oregon, and Washington, respectively, and the denominator, $\left[10\left(A_{CA}+A_{OR}\right)+9A_{WA}\right]$ is a scaling constant to ensure that $f\left(p_i\right)$ is a proper probability density function. Then, for a sample of size *n*, the inclusion density function is $\pi\left(p_i\right)=f\left(p_i\right)/n$.

The estimator in eq. (1) reduces to a weighted average of the latitude, elevation or temperature of the plots that contain either seedlings or trees of the species of interest. The weight is 1 for plots in California and Oregon, and 10/9 for plots in Washington, thus accounting for the different sampling intensity. In this analysis, nonresponse is treated as if it were missing at random within the range of the species ([4], p. 41). However, the bias introduced by non-response is likely to be negligible. The non-response rate is relatively low. More importantly, the parameter of interest is the difference between the means of the range of seedlings and mature trees. A bias in the estimator of the difference would require a different non-response process affecting the range of seedlings and trees, which is unlikely given the large overlap between those ranges.

An approximate estimator of the variance of this ratio estimator is ([1], eq. 5.6.10):

$$\hat{V}\left(\hat{\mu}_{kd}\right)\approx\frac{1}{\left|\hat{D}_{kd}\right|^2}\left[\hat{v}\left(\hat{\tau}_{kd}\right)+\hat{\mu}_{kd}^2\hat{v}\left(\left|\hat{D}_{kd}\right|\right)-2\hat{\mu}_{kd}\hat{c}\left(\hat{\tau}_{kd},\left|\hat{D}_{kd}\right|\right)\right] \tag{4}$$

where $\hat{\tau}_{kd}=\sum_{i=1}^{n}I_{kd}\left(p_i\right)y\left(p_i\right)/\pi\left(p_i\right)$ is the numerator of eq. (1), and $\hat{v}(\cdot)$ and $\hat{c}(\cdot,\cdot)$ are the estimated variance and covariance functions, respectively. For arbitrary estimators $\hat{x}$ and $\hat{z}$, they are:

$$\hat{v}\left(\hat{x}\right)=\frac{1}{n\left(n-1\right)}\sum_{i=1}^{n}\left(\frac{x\left(p_i\right)}{f\left(p_i\right)}-\hat{x}\right)^2 \tag{5}$$

$$\hat{c}\left(\hat{x},\hat{z}\right)=\frac{1}{n\left(n-1\right)}\sum_{i=1}^{n}\left(\frac{x\left(p_i\right)}{f\left(p_i\right)}-\hat{x}\right)\left(\frac{z\left(p_i\right)}{f\left(p_i\right)}-\hat{z}\right) \tag{6}$$

These estimators and the associated confidence intervals are obtained by treating the sample as if it had been selected using independent random sampling, instead of a spatially balanced design. Because a balanced design is more efficient than an independent sampling design in the presence of spatial correlation, those variance estimators would tend to be conservative and overstate the sampling variance [5].

For each species, we estimated the difference in the mean elevation, latitude or annual temperature for the range of seedlings minus that for the range of mature trees as the difference between their respective domain ratio estimators:

$$\hat{\delta}_k=\hat{\mu}_{kS}-\hat{\mu}_{kT} \tag{7}$$

The approximate variance of $\hat{\delta}_k$, using a Taylor linearization method ([6], eq. 6.9.1), is:

$$\hat{V}\left(\hat{\delta}_k\right)\approx\hat{V}\left(\hat{\mu}_{kS}\right)+\hat{V}\left(\hat{\mu}_{kT}\right)-\frac{2}{\left|\hat{D}_{kS}\right|\left|\hat{D}_{kT}\right|}\begin{bmatrix}\hat{c}\left(\hat{\tau}_{kS},\hat{\tau}_{kT}\right)+\hat{\mu}_{kS}\hat{\mu}_{kT}\hat{c}\left(\left|\hat{D}_{kS}\right|,\left|\hat{D}_{kT}\right|\right)-\\ \hat{\mu}_{kT}\hat{c}\left(\hat{\tau}_{kS},\left|\hat{D}_{kT}\right|\right)-\hat{\mu}_{kS}\hat{c}\left(\hat{\tau}_{kT},\left|\hat{D}_{kS}\right|\right)\end{bmatrix} \tag{8}$$

We estimated a 95% confidence interval as:

$$\hat{\delta}_k\pm z_{0.975}\left[\hat{V}\left(\hat{\delta}_k\right)\right]^{1/2} \tag{9}$$

where $z_{0.975}$ is the 97.5 percentile of the normal distribution.

For each species, we estimated the 5th and 95th percentiles of the distribution of temperature for seedlings and mature trees. First, we estimated the population distribution function ([7], p.69), and then calculated its inverse evaluated at 0.05 and 0.95. We computed the difference between

the estimator for seedlings minus that for trees and obtained 95% confidence intervals by the percentile bootstrap method ([8], Chapter 6).

Estimation of mean differences across all species

The variance of the estimator for the individual species effect differed widely among species, mostly due to large differences in sample sizes. Further, because the estimators were calculated from the same sample, they are correlated. To deal with the unequal variance and lack of independence, we estimated the mean difference across all species using a generalized least squares estimator:

$$\hat{\Delta} = \left(\mathbf{c}^{\mathbf{T}}\mathbf{\Sigma}^{\mathbf{-1}}\mathbf{c}\right)^{\mathbf{-1}}\mathbf{c}^{\mathbf{T}}\hat{\boldsymbol{\delta}} \qquad (10)$$

where $\hat{\boldsymbol{\delta}}$ is a vector of the individual species differences, $\hat{\delta}_k$, $\mathbf{c}$ is a vector of 1s of the same length as $\hat{\boldsymbol{\delta}}$ (46) and $\mathbf{\Sigma}$ is the covariance matrix of $\boldsymbol{\delta}$. The variance of this estimator is:

$$V\left(\hat{\Delta}\right) = \left(\mathbf{c}^{\mathbf{T}}\mathbf{\Sigma}^{\mathbf{-1}}\mathbf{c}\right)^{\mathbf{-1}} \qquad (11)$$

We estimated the variance $\mathbf{\Sigma}$ from the sample. An analytical expression for $\mathbf{\Sigma}$ would be cumbersome and difficult to obtain, so we used the bootstrap (8, Chapter 6). We took a sample with replacement from the 33,674 plots and computed $\hat{\boldsymbol{\delta}}$ (eq. (7)) and the estimates of the difference in the 5th and 95th percentiles. We repeated this process 20,000 times and calculated the covariance matrices of the bootstrap replications.

References

1. Särndal CE, Swensson B, Wretman J (1992) Model Assisted Survey Sampling. New York: Springer-Verlag. 694 p.

2. Cordy C (1993) An extension of the Horvitz-Thomson theorem to point sampling from a continuous population. Stat Probab Lett 18: 353-362.

3. Gregoire TG, Valentine HT (2008) Sampling strategies for natural resources and the environment. Boca Raton: Chapman & Hall / CRC. 496 p.

4. Bechtold WA, Scott CT (2005) The forest inventory and analysis plot design. In Bechtold WA, Patterson PL, editors. The enhanced Forest Inventory and Analysis Program – national sampling design and estimation procedures. Gen. Tech. Rep. SRS-80. Asheville: US Forest Service. pp. 27-42.

5. Stevens DL, Olsen AR (2003) Variance estimation for spatially balanced samples of environmental resources. Environmetrics 14: 593-610.

6. Wolter KM (1985) Introduction to Variance Estimation. New York: Springer-Verlag. 427 p.

7. Fuller WA (2009) Sampling Statistics. Hoboken: Wiley. 454 p.

8. Shao J, Tu D. (1995) The Jackknife and Bootstrap. New York: Springer-Verlag. 517 p.
